# Impact of Long Non-Coding RNA *HOTAIR* Genetic Variants on the Susceptibility and Clinicopathologic Characteristics of Patients with Urothelial Cell Carcinoma

**DOI:** 10.3390/jcm8030282

**Published:** 2019-02-26

**Authors:** Min-Che Tung, Yu-Ching Wen, Shian-Shiang Wang, Yung-Wei Lin, Jyh-Ming Chow, Shun-Fa Yang, Ming-Hsien Chien

**Affiliations:** 1 Graduate Institute of Clinical Medicine, College of Medicine, Taipei Medical University, Taipei 11031, Taiwan; tungminche@yahoo.com.tw; 2 Department of Surgery, Tungs’ Taichung Metro Harbor Hospital, Taichung 43304, Taiwan; 3 Department of Urology, Wan Fang Hospital, Taipei Medical University, Taipei 11031, Taiwan; s811007@yahoo.com.tw (Y.-C.W.); highwei168@gmail.com (Y.-W.L.); 4 Department of Urology, School of Medicine, College of Medicine, Taipei Medical University, Taipei 11031, Taiwan; 5 Division of Urology, Department of Surgery, Taichung Veterans General Hospital, Taichung 00407, Taiwan; sswdoc@vghtc.gov.tw; 6 School of Medicine, Chung Shan Medical University, Taichung 40201, Taiwan; 7 Institute of Medicine, Chung Shan Medical University, Taichung 40201, Taiwan; 8 Division of Hematology and Medical Oncology, Department of Internal Medicine, Wan Fang Hospital, Taipei Medical University, Taipei 11031, Taiwan; chow0803@yahoo.com.tw; 9 Department of Medical Research, Chung Shan Medical University Hospital, Taichung 40201, Taiwan; 10 Department of Medical Education and Research, Wan Fang Hospital, Taipei Medical University, Taipei 40201, Taiwan

**Keywords:** long non-coding RNA, HOX antisense intergenic RNA, polymorphism, susceptibility, clinicopathologic characteristics, urothelial cell carcinoma

## Abstract

Increasing evidence shows that dysregulated expression of long non-coding (lnc)RNAs can serve as diagnostic or prognostic markers in urothelial cell carcinoma (UCC), the most common pathological type of bladder cancer. lncRNA *HOX transcript antisense RNA* (*HOTAIR*) was shown to promote tumor progression and be associated with a poor prognosis in multiple cancers including bladder cancer. Polymorphisms of *HOTAIR* were recently linked to a predisposition for diverse malignancies. Herein we conducted a case-control study to evaluate whether genetic polymorphisms of *HOTAIR* were associated with UCC risk and clinicopathologic characteristics. Four loci (rs920778 T>C, rs1899663 G>T, rs4759314 A>G, and rs12427129, C>T) of *HOTAIR* were genotyped by a TaqMan allelic discrimination method in 431 cases and 862 controls. We found that female patients who carried AG + GG genotype of rs4759314 were associated with an increased UCC risk after controlling for age and tobacco consumption (adjusted odds ratio (AOR) = 1.92, 95% confidence interval (CI): 1.01–3.64, *p* = 0.047) and a lower overall survival rate (*p* = 0.008). Moreover, patients with a smoking habit or younger age (≤65 years), who had at least one T allele of *HOTAIR* rs12427129 were at a higher risk of developing advance tumor T satge (*p* = 0.046), compared to those patients with CC homozygotes. In contrast, rs920778 C allele carriers were negatively correlated with the development of lymph node metastasis (OR = 0.51, 95% CI: 0.28–0.94, *p* = 0.031). Further analyses of clinical datasets revealed correlations of the expression of *HOTAIR* with tumor metastasis and a poor survival rate in patients with UCC. Our results verified the diverse impacts of *HOTAIR* variants on UCC susceptibility and clinicopathologic characteristics.

## 1. Introduction

Urothelial cell carcinoma (UCC) accounts for about 90% of all bladder cancers. In Taiwan, the mortality rates of bladder cancer patients respectively ranked 13th and 12th among all cancer deaths in females and males [1]. UCC is a smoking-related cancer, and similar to other such cancers, UCC has a high rate of somatic mutations and single-nucleotide polymorphisms (SNPs) [2,3]. A large number of genes are reported to be associated with susceptibility and development of various cancer types containing single-nucleotide polymorphisms (SNPs). These SNPs are located in diverse regions of the DNA, including promoters, exons, introns, 5′- and 3′-untranslated regions (UTRs), and intergenic regions, and affect gene expressions through different mechanisms [4]. For example, SNPs in promoter regions can modulate gene expressions by influencing binding of transcription factors (TFs) and promoter activities [5,6]. The exonal SNPs affect cancer susceptibility through suppressing gene transcription or translation. The intronic SNPs are reported to generate splice variants of transcripts and enhance or block the binding and function of long non-coding RNAs (lnc)RNAs [4]. SNPs located in intergenic regulatory sequences, such as enhancers, might affect the binding of TFs to an enhancer, and further affect the transcription of RNA transcripts, which could then influence disease-related pathways and trigger disease phenotypes [7]. SNPs in the 5′-UTR are reported to influence translation [8], whereas SNPs in the 3′-UTR affect micro (mi)RNA binding [9]. From a clinical perspective, SNPs are important and are potential diagnostic, prognostic, and therapeutic biomarkers in multiple cancer types, including UCC [4,10].

LncRNAs are defined as RNA transcripts longer than 200 nucleotides that lack significant open reading frames, and which are unable to code for proteins. LncRNAs are a major class of RNA molecules which play an important role in multiple cancers [11]. Deeper understanding of the roles of lncRNAs in tumor growth, progression, and prognosis could contribute to a large number of potential clues for developing novel therapeutic approaches for cancers. Recently, increasing evidence showed that lncRNAs regulate various cellular behaviors, such as tumorigenesis, tumor invasion, and metastasis through transcriptional, post-transcriptional, and epigenetic regulation of cancer-associated genes [12]. Therefore, because aberrant expression patterns of lncRNAs are correlated with the diagnosis and prognosis of cancers, they can serve as predictors of patient outcomes. For example, lncRNA *H19* expression is upregulated and was correlated with TNM stages in patients with gastric cancer, and thus can serve as a potential non-invasive diagnostic biomarker in these patients [13]. In cervical cancer, lncRNA *maternally expressed gene 3 (MEG3)* was reported to be conversely associated with the tumor size and lymphatic metastasis, indicating its critical role as a tumor suppressor and a potential therapeutic candidate. Overexpression of *MEG3* in cervical cancer cells attenuated the miR-21-5p expression level, causing inhibition of proliferation, and induction of apoptosis [14].

In urothelial bladder carcinoma, approximately 50 lncRNAs were found to be aberrantly expressed and were shown to have prognostic and diagnostic values [15]. For example, *metastasis-associated lung adenocarcinoma transcript-1 (MALAT1)* was reported to associate with lymph node metastasis and poor overall survival. High expression of *X-inactive specific transcript (XIST)* was shown to correlate with larger tumor size and higher tumor/node/metastasis (TNM) stage. Among these lncRNAs, a recently discovered lncRNA, *HOX antisense intergenic RNA* (*HOTAIR*), was upregulated in the urine of UCC patients with high-grade muscle-invasive disease (i.e., pT2–pT4) [16] and was correlated with a poor prognosis of recurrence-free survival [17,18]. Moreover, knockdown of *HOTAIR* in UCC cell lines inhibited their in vitro migratory, invasive, and proliferative abilities and the epithelial-to-mesenchymal transition (EMT) [16]. *HOTAIR* plays an oncogenic role in UCC, similar to what it does in many other cancer types [12].

*HOTAIR* arises from transcription of the antisense strand of the *HOXC* gene, which is specifically situated between *HOXC11* and *HOXC12* on chromosome 12q13.13 [19]. As to the relationship between *HOTAIR* genetic variants and cancer, multiple SNPs of *HOTAIR* were recently identified as potential cancer susceptibility loci, and linked to the risks of several human cancer types, including breast, ovarian, esophageal squamous cell, gastric, cervical, and colorectal cancers [20,21,22]. However, the effects of *HOTAIR* gene polymorphisms on the risk of UCC remain unexplored. Accordingly, we selected four intronic SNPs (rs920778, rs1899663, rs4759314, and rs12427129) which were recently identified to be associated with cancer risk or development [23,24] and conducted the present case-control study which included 431 UCC patients and 862 healthy controls, in an attempt to investigate the roles of *HOTAIR* SNPs in the risk and clinical characteristics of UCC in a Taiwanese population.

## 2. Materials and Methods

### 2.1. Study Populations, Ethics, and Consent

The study groups of this case-control study comprised 862 cancer-free controls and 431 urothelial cell carcinoma (UCC) patients recruited from Taichung Veterans General Hospital (Taichung, Taiwan). For the control group, we randomly chose the individuals who received routine physical examinations in this hospital and had no self-reported history of cancer at any site. The controls frequently matched to cases on gender. All participants were of Asian ethnic background and from the same geographic area. The clinical staging of UCC patients was carried out at the time of diagnosis following guidelines of the TNM staging system of the American Joint Committee on Cancer (AJCC). Both the case and control groups were asked about their exposure history to tobacco consumption through interviewer-administered questionnaires. The tobacco consumption is categorized in “never-user” and “ever-user (current and former user)” at the time of diagnosis. The study protocol was approved by the Institutional Review Board of Taichung Veterans General Hospital (IRB no. CF11094), and informed written consent was obtained from each individual before initiation of the study. Venous blood from each participant was taken in an ethylenediaminetetraacetic acid (EDTA)-containing tube, immediately centrifuged, and then stored at −80 °C.

### 2.2. Genomic DNA Extraction and Selection of HOTAIR SNPs

Total DNA was extracted from peripheral blood of each participant using a QIAamp DNA Blood Mini Kit (Qiagen, Valencia, CA, USA) according to the manufacturer’s protocols. In total, four intronic SNPs of *HOX transcript antisense RNA* (*HOTAIR*) (rs920778 T>C, rs1899663 G>T, rs4759314 A>G, and rs12427129 C>T) were chosen in this study based on the International HapMap Project and previous studies [23,24,25,26]. These four *HOTAIR* SNPs were selected due to these SNPs being well-defined, and have been widely evaluated in their association with cancer susceptibility in a broad range of cancers such as oral, lung, cervical, and liver cancers in the Han Chinese population from Taiwan and Mainland China [23,24,26,27]. The minor allele frequencies of these four SNPs were all ≥5%. Moreover, a meta-analysis showed that a significant association between *HOTAIR* rs920778, or rs4759314 SNPs and cancer susceptibility in different cancer types [25] and these two SNPs have been reported to affect the expression of *HOTAIR* in different cancer types [27,28,29,30].

### 2.3. Genotyping of HOTAIR SNPs

Allelic discrimination of *HOTAIR* rs920778 (assay ID: C_9162435_20), rs1899663 (assay ID: C_2104251_20), rs4759314 (assay ID: C_27930754_10), and rs12427129 (assay ID: C_2104247_10) SNPs was assessed using the TaqMan SNP Genotyping Assay with an ABI StepOnePlus™ Real-Time Polymerase Chain Reaction (PCR) System and further evaluated with SDS version 3.0 software (Applied Biosystems, Foster City, CA, USA) as described previously [31].

### 2.4. HOTAIR Expression Profiles of Bladder Cancer Patients from Gene Expression Omnibus (GEO) and the Cancer Genome Atlas (TCGA) Data Sets

We used TCGA dataset (https://tcga-data.nci.nih.gov/) to obtain the *HOTAIR* normalized expression data and the associated clinical data, which corresponds to the urothelial bladder carcinoma dataset. Box plots for *HOTAIR* expression values were created with respect to the lymph node status (N0: 209 and N1: 38) and Metastasis (M0: 169 and M1: 10). *HOTAIR* expression from gene expression omnibus (GEO) (http://www.ncbi.nlm.nih.gov/geo/) dataset (GSE13507) was downloaded and processed. We analyzed the 165 primary urothelial bladder carcinoma samples for the Kaplan-Meier survival analysis. The probe ID of *HOTAIR* we used is ILMN_1904054. If the probe intensity of the sample was greater than or equal to 7.06, we delimited the expression of *HOTAIR* is high.

### 2.5. Statistical Analysis

The adjusted odds ratios (AORs) with 95% confidence intervals (CIs) of associations between genotype frequencies and clinicopathologic characteristics, were estimated by multivariable logistic regression models, after controlling for age, gender and tobacco consumption. The associations between genotype frequencies and clinicopathologic characteristics were also stratified by gender, age and smoking status. Two different genetic models (dominant model and co-dominant/genotypic model) were used to analyze the effect of SNPs. The patients were followed to calculate the overall survival between primary surgery and death, or end of the study (June 30, 2018). All-cause of death was examined by a Kaplan-Meier survival curve. A *p*–value <0.05 was considered significant. All data were analyzed with Statistical Analytical System (SAS Institute, Cary, NC, USA) software (version 9.1) for Windows.

## 3. Results

### 3.1. Population Characteristics

The study group comprised 431 pathologically confirmed UCC patients (272 males and 159 females), with a mean age of 68.6 ± 11.8 years, and 862 cancer-free controls (566 males and 296 females), with a mean age of 57.2 ± 10 years. There was a significant difference in the distribution of age between these two groups (*p* < 0.001). Our UCC patients were predominantly an older age (>65 years, 61.5%), while the controls were younger (≤65 years, 80.9%) (Table 1). In contrast, no significant differences in the distributions of sex (*p* = 0.365) or tobacco use (*p* = 0.113) were observed between cases and controls. Most patients were suffering from superficial tumors (stage pTa–pT1) without lymph node or distal metastasis, and these clinical characteristics were compatible with observations from other researchers [32,33].

### 3.2. Associations between HOTAIR Gene Polymorphisms and UCC Susceptibility in Different Genders

To examine the possible association of *HOTAIR* gene polymorphisms with the risk of developing UCC, the genotype frequencies of four intronic SNPs (viz., rs920778, rs1899663, rs4759314, and rs12427129) were investigated in the entire population we recruited (Table 2). Genotypic distributions of *HOTAIR* rs920778, rs1899663, rs4759314, and rs12427129 conformed to Hardy–Weinberg equilibrium (*p* = 0.119, χ2 value: 2.425; *p* = 0.172, χ2 value: 1.863; *p* = 0.604, χ2 value: 0.269 and *p* = 0.068, χ2 value: 3.324, respectively). To reduce the possible interference of confounding variables, we used AORs and 95% CIs which were estimated by multivariable logistic regression models after adjusting each comparison for the covariates described above. Among these tested SNPs, no significant differences in genotype distributions between UCC patients and controls were found (Table 2). We further divided the tested population by sex, and found that female UCC patients carrying the *HOTAIR* rs4759314 AG + GG genotype (AG + GG vs. AA: AOR, 1.92; 95% CI, 1.01–3.64; *p* = 0.047] were associated with an increased risk of UCC (Table 3), while no associations were found in any male UCC patients (Table A1).

### 3.3. Relationships of Clinicopathological Characteristics with HOTAIR Genetic Polymorphisms in UCC Patients

Subsequently, we further analyzed genotype frequencies of individual polymorphisms in relation to the clinicopathological status, such as the cancer stage, primary tumor size, lymph node involvement, metastatic status, and histopathologic grading, in UCC patients. We divided overall UCC patients into two subgroups, including patients who had homozygous wild-type (WT) alleles and those who had at least one polymorphic allele. The comparison showed that patients with at least one minor allele (TC and CC) of rs920778 exhibited a significantly (*p* = 0.031) lower 0.51-fold risk (95% CI, 0.28–0.94) of developing lymph node metastasis compared to their corresponding WT homozygotes (Table 4), while no associations were found in *HOTAIR* rs1899663, rs4759314 and rs12427129 with clinicopathologic characteristics in UCC patients (Table A2). Cigarette smoking was reported to increase the risk of developing high-grade superficial and invasive bladder cancer [34]. In this study, we further divided our recruited UCC patients into smoking and non-smoking groups, and further investigated the difference between *HOTAIR* SNPs and the UCC clinicopathological status in these two groups. The 131 UCC smokers harboring the rs12427129 CT/TT SNP had a higher risk (4.62-fold; 95% CI, 1.03–20.78) of developing advance tumor T stage (*p* = 0.046) than did WT patients (Table 5). In addition to smoking, the association between aging and cancer is well exemplified by bladder cancer. Previous reports simply defined “elderly” as those bladder cancer patients aged over 65 years, with no additional clinical information to help better define this particular subset of older adults [35]. Herein, we observed that among 166 younger UCC patients aged ≤65 years, those carrying at least one polymorphic T allele of *HOTAIR* rs12427129 showed a significantly higher risk of having a larger primary tumor (OR = 3.57; 95% CI, 1.03–12.44, *p* = 0.046) than those carrying the WT gene (Table 6). However, no associations were found in *HOTAIR* rs920778, rs1899663 and rs4759314 in patients with UCC, stratified by age and smoking status (Table A3).

### 3.4. Associations between HOTAIR Gene Polymorphisms and the overall survival of UCC Patients

**Figure 1:**
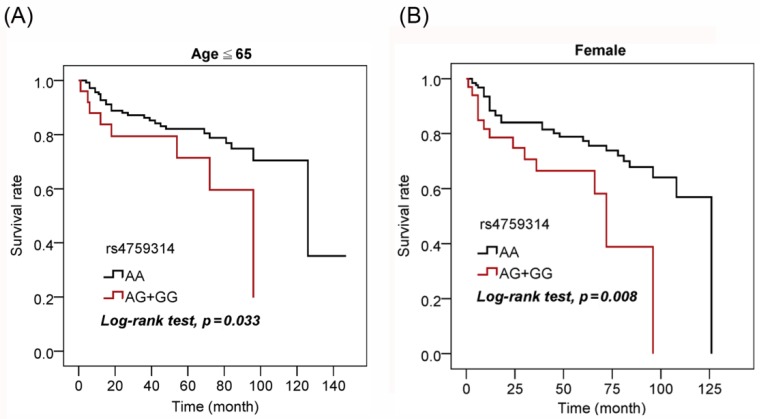
Association of rs4759314 with survival of urothelial cell carcinoma (UCC) patients. Kaplan-Meier analysis of correlations between rs4759314 genotypes and overall survival of 166 younger (aged ≤65 years) UCC patients (**A**) and 159 female UCC patients (**B**).

Next, we further assessed the prognostic value of *HOTAIR* polymorphisms in UCC patients. An analysis based on a Kaplan-Meier curve revealed that G carriers of rs4759314 presented worse overall survival than those with the WT gene in the younger patient (aged ≤65 years) subgroup (*p* = 0.033; Figure 1A) and female subgroup (*p* = 0.008; Figure 1B).

### 3.5. Clinical Relevance of HOTAIR Levels in Urothelial Bladder Cancer Patients Obtained from TCGA and GEO Databases

**Figure 2:**
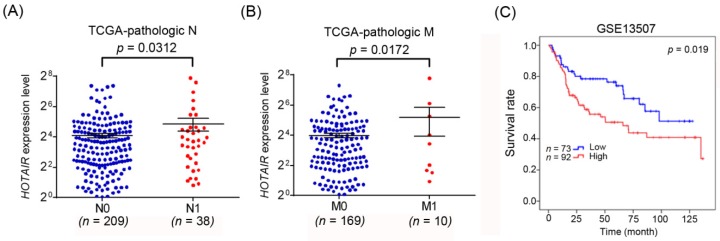
High expression of *HOTAIR* in urothelial bladder cancer tissues is associated with patients with tumor metastasis and poor prognoses. (**A**,**B**) *HOTAIR* gene expression levels in urothelial bladder cancer tissues from TCGA were compared according to the lymph node metastasis status (N stages) (**A**) and distal metastasis status (M stages) (**B**). (**C**) A Kaplan-Meier plot of overall survival of 165 patients with urothelial bladder cancer (GSE13507) stratified by *HOTAIR* expression. A log-rank test was used to examine between-group differences.

Considering the potential effects of *HOTAIR* polymorphic genotypes and higher *HOTAIR* expression levels, we further analyzed correlations of *HOTAIR* expression with patient clinicopathological characteristics and the survival rate. As shown in Figure 2A,B, significantly higher *HOTAIR* transcripts were observed in tumors of patients with lymph node and distal metastases, compared to patients without tumor metastasis. Furthermore, 165 urothelial bladder cancer cases were also analyzed from the GEO database (GSE13507), and we found that patients with *HOTAIR*^high^ tumors had shorter overall survival times compared with those with *HOTAIR*^low^ tumors (*p* = 0.019; Figure 2C). Taken together, the above clinical data suggest that *HOTAIR* genetic variants may affect *HOTAIR* expression levels and subsequently modulate UCC progression.

## 4. Discussion

In UCC of the bladder, many cancer-related lncRNAs have been shown to exert prognostic or diagnostic value. For instance, the *MALAT1* expression level was associated with lymph node metastasis and poor survival rate of UCC patients. LncRNA *urothelial cancer-associated 1 (UCA1)* showed the diagnostic value for UCC of the bladder [15]. Both in vivo and in vitro studies have shown that *MALAT1* can promote migration and metastasis of bladder cancer through inducing EMT [36,37]. Similar to *MALAT1*, *UCA1* was also reported to induce increase of the migratory and invasive abilities of bladder cancer cells by inducing EMT [38]. In contrast to oncogenic lncRNAs, *MEG3* was reported to be a tumor suppressor in UCC of the bladder and downregulation of *MEG3* expression was associated with lower recurrence-free survival [39].

*HOTAIR*, a transacting lncRNA, is reported to be a scaffold through which polycomb repressive complex 2 (PRC2) and lysine-specific demethylase 1 (LSD1) complexes are recruited to target genes which respectively result in methylation and demethylation of H3K27 and H3K4, which together epigenetically regulate various downstream genes. The overexpression of *HOTAIR* in various cancers, including UCC, indicates its important role in their development [19]. The oncogenic roles of *HOTAIR* in UCC were previously extensively studied [16,17,18,40], while epidemiological focus on UCC susceptibility and clinicopathologic characteristics conferred by genetic variants on loci of *HOTAIR* has rarely been investigated.

In the present study, we performed a case-control study to evaluate associations between four SNPs in the *HOTAIR* gene and UCC risk. We observed that SNP rs4759314 A > G was significantly associated with an increased UCC risk and poor overall survival in younger and female subjects. This *HOTAIR* rs4759314 polymorphism was also reported to be associated with a risk of gastric cancer in a Chinese population. The rs4759314 polymorphism is located in an intronic promoter region (intron 1), which was found to influence the activity of this promoter and expressions of the *HOTAIR* gene and a nearby gene, *HOXC11*. In gastric cancer cases, increased *HOTAIR* expression was associated with G allele carriers [30]. In a meta-analysis showed that *HOTAIR* rs4759314 polymorphism was a risk factor only for gastric cancer, but not for lung, breast, colorectal, and esophageal cancer in Chinese populations [25]. Based on these observations, we suggest that the impact of rs4759314 polymorphism is dependent on different cancer types.

In addition to rs4759314, previous reports indicated that there might be a potential enhancer in the *HOTAIR* intron 2 region containing the rs920778 SNP. It was reported that the functional effect of the *HOTAIR* rs920778 SNP T variant allele was to promote enhancer activity and expression of *HOTAIR* in esophageal squamous cell carcinoma cells, gastric cancer, and cervical cancer cells as well as in tissues of these cancer types in a Chinese population [27,28,29].

Cancer patients with these three cancer types who harbored the *HOTAIR* rs920778T allele were significantly associated with an elevated risk or an advanced TNM stage of these cancers compared to those with the C allele in a Chinese population [27,28,29]. Recently, gene polymorphisms in rs920778 were also found to significantly increase one’s susceptibility to lung cancer in a Chinese lung cancer population [24]. About the analysis stratified by ethnicity, there is a meta-analysis which showed that a significant association between rs920778 SNPs and cancer susceptibility was observed in Asians under dominant, recessive, homozygous, and heterozygous models. This association was most compelling under recessive model (TT vs. TC+CC). The *HOTAIR* rs920778T allele thus emerged as a potential genetic marker for increased cancer susceptibility especially in Asians [25]. In our current study, UCC patients with the *HOTAIR* polymorphic rs920778 TC + CC genotypes exhibited a significantly lower risk of developing lymph node metastasis compared to the rs920778 TT homozygous polymorphism. By virtue of the rs920778T allele being correlated with significantly increased *HOTAIR* RNA expression in tissues from different cancer types, we speculated that the rs920778C allele acts as a protective factor to prevent lymph node metastasis of UCC through decreasing *HOTAIR* expression in UCC tissues. According to our previous assessment of the enhancer region of human *HOTAIR*, we identified that the rs920778 position harbors a putative binding motif of PRDI-BF1 and RIZ domain containing 14 (PRDM14) [23], a site-specific transcriptional activator or repressor that acts as a tumor suppressor or oncogene [41]. It was reported that lncRNA *Tsix* can facilitate PRDM14’s binding to intron 1 of lncRNA of *Xist*, thereby repressing *Xist* expression [42]. Hence, relationships among the *HOTAIR* rs920778 polymorphism, PRDM14-intron 2 binding, and *HOTAIR* expression remain unclear and are worthy of further investigation.

The impacts of *HOTAIR* gene polymorphisms on the risk or development of UCC may be underestimated in such a complex scenario. Environmental factors such as smoking are important risk factors for developing bladder cancer [43]. In our study, SNP-environment interactions for rs12427129 and the smoking status were found to enhance the risk of developing larger UCC tumors. Previous reports indicated that higher *HOTAIR* expression was observed in lung cancer patients with the tobacco smoking habit compared to those who did not smoke [24]. Moreover, cigarette smoke extract (CSE) was reported to induce interleukin-6 and further turn on stat3-mediated *HOTAIR* expression in human bronchial epithelial cells [44]. CSE was also reported to induce activation of the transcriptional co-activator p300 in human tracheal smooth muscle cells [45], and prior studies predicted that rs12427129 polymorphisms might affect the binding of p300 to *HOTAIR* [26]. Furthermore, from the GTEx database, alteration of *HOTAIR* expression was only observed in skin tissues, but not in bladder or other tissues of individuals who carry the polymorphic allele of rs12427129 [46]. Based on the above annotations, we speculated that tobacco carcinogens might alter *HOTAIR* expression dependent on the presence of rs12427129 polymorphisms, and this issue should be further addressed in the future. In addition to four *HOTAIR* SNPs we investigated in this study, Wang et al. recently reported that rs874945 SNP, located in the 3′-UTR of the *HOTAIR* gene, is significantly associated with the risk of bladder cancer. The results from this study indicated that the effects of the *HOTAIR* rs874945 variant on bladder cancer risk might be modulated by environmental exposures such as tobacco smoking [47]. The impacts of *HOTAIR* rs874945 polymorphisms combined with smoking status on tumorigenesis of urothelial bladder cancer are worthy of being further addressed in our study cohort.

Our study is not without limitations. The limitation of this study is the lacking in mRNA collection from peripheral blood of our study cohort. Although previous studies have indicated that *HOTAIR* rs4759314 and rs920778 polymorphisms can affect the expression of *HOTAIR* in different cancer types [27,28,29,30], we cannot validate the association between genetic variants of *HOTAIR* with expression level of *HOTAIR* in UCC. In our future study, the mRNA and DNA should be extracted simultaneously from the same samples to further validate this issue. Further limitation of the study is the lacking of another independent cohort to double-check our current results. Our sample size had at least an 80% power to detect a 1.5-fold increased risk in susceptibility to UCC associated with *HOTAIR* genetic polymorphisms. However, the negative results could be explained by too low power of the study. 

Moreover, this study is restricted to the Taiwanese population (of Asian or Chinese ethnicity), so it is uncertain whether or not these results can be generalized to other populations. Hence, future studies of these SNPs in different UCC cohorts or different ethnic populations are necessary to confirm our results.

## 5. Conclusions

Despite our best efforts, a significant proportion of UCC patients will eventually develop advanced disease, and we do not currently have reliable tools to predict who those patients are. In this study, we first identified the diverse allelic effects of *HOTAIR* SNPs (rs920778, rs4759314, and rs12427129) which contribute to the susceptibility and clinicopathologic development of UCC in a Taiwanese population. These results can lead to a better understanding of risk and early detection of UCC. Additional studies should focus on investigating the functional activities of these polymorphisms analyzed above and their effects on UCC progression.

## Figures and Tables

**Table 1:** The distributions of demographical characteristics in 431 patients with UCC and 862 controls.

Variable	Controls (*n* = 862) *n* (%)	Patients (*n* = 431) *n* (%)	*p*-Value
Age (years)			
mean ± S.D.	57.2 ± 10	68.6 ± 11.8	*p* < 0.001
≤65	697 (80.9%)	166 (38.5%)	*p* < 0.001
>65	165 (19.1%)	265 (61.5%)	
Gender			
Male	566 (65.7%)	272 (63.1%)	0.365
Female	296 (34.3%)	159 (36.9%)	
Tobacco consumption			
No	562 (65.2%)	300 (69.6%)	0.113
Yes	300 (34.8%)	131 (30.4%)	
Stage			
Non-muscle invasive tumor		235 (54.5%)	
Muscle invasive tumor		196 (45.5%)	
Tumor T Status			
Ta		90 (16.5%)	
Tcis		19 (4.4%)	
T1		145 (33.6%)	
T2		52 (12.1%)	
T3		107 (24.8%)	
T4		37 (8.6%)	
Lymph Node Status			
N0		380 (88.2%)	
N1		13 (3.0%)	
N2		33 (7.6%)	
N3		5 (1.2%)	
Metastasis			
M0		417 (96.8%)	
M1		14 (3.2%)	
Histopathologic Grading			
Low grade		53 (12.3%)	
High grade		378 (87.7%)	

S.D., standard deviation.

**Table 2:** Genotype distributions of *HOTAIR* gene polymorphisms in 862 controls and 431 patients with urothelial cell carcinoma.

Variable	Controls (*n* = 862) *n* (%)	Patients (*n* = 431) *n* (%)	OR (95% CI)	AOR (95% CI)
rs920778				
TT	447 (51.9%)	217 (50.3%)	1.00 (reference)	1.00 (reference)
TC	360 (41.8%)	176 (40.8%)	1.01 (0.79–1.28)	0.96 (0.73–1.27)
CC	55 (6.4%)	38 (8.8%)	1.42 (0.91–2.22)	1.60 (0.96–2.68)
TC + CC	415 (48.1%)	214 (49.7%)	1.06 (0.84–1.34)	1.04 (0.80–1.35)
rs1899663				
GG	550 (63.8%)	265 (61.5%)	1.00 (reference)	1.00 (reference)
GT	285 (33.1%)	148 (34.3%)	1.08 (0.84–1.38)	0.95 (0.71–1.25)
TT	27 (3.1%)	18 (4.2%)	1.38 (0.75–2.56)	1.85 (0.92–3.73)
GT + TT	312 (36.2%)	166 (38.5%)	1.10 (0.87–1.40)	1.01 (0.77–1.32)
rs4759314				
AA	727 (84.3%)	363 (84.2%)	1.00 (reference)	1.00 (reference)
AG	128 (14.8%)	67 (15.5%)	1.05 (0.76–1.45)	1.07 (0.74–1.54)
GG	7 (0.8%)	1 (0.2%)	0.29 (0.04–2.33)	0.59 (0.06–5.58)
AG + GG	135 (15.7%)	68 (15.8%)	1.01 (0.73–1.39)	1.05 (0.73–1.51)
rs12427129				
CC	691 (80.2%)	350 (81.2%)	1.00 (reference)	1.00 (reference)
CT	167 (19.4%)	76 (17.6%)	0.90 (0.67–1.21)	1.00 (0.71–1.41)
TT	4 (0.5%)	5 (1.2%)	2.47 (0.66–9.25)	2.23 (0.44–11.23)
CT + TT	171 (19.8%)	81 (18.8%)	0.94 (0.70–1.26)	1.03 (0.74–1.44)

The odds ratio (OR) and 95% confidence interval (CI) were estimated by logistic regression models. The adjusted OR (AOR) and the 95% CI were estimated by multivariable logistic regression models after controlling for age, gender, and tobacco consumption.

**Table 3:** Genotype distributions of *HOTAIR* gene polymorphisms among 455 female subjects.

Variable	Controls (*n* = 296), *n* (%)	Patients (*n* = 159), *n* (%)	OR (95% CI)	AOR (95% CI)
rs920778				
TT	155 (52.4%)	79 (49.7%)	1.000 (reference)	1.00 (reference)
TC	120 (40.5%)	61 (38.4%)	1.00 (0.66–1.50)	1.03 (0.62–1.71)
CC	21 (7.1%)	19 (11.9%)	1.78 (0.90–3.49)	1.96 (0.84–4.61)
TC + CC	141 (47.6%)	80 (50.3%)	1.11 (0.76–1.64)	1.16 (0.72–1.88)
rs1899663				
GG	191 (64.5%)	95 (59.7%)	1.00 (reference)	1.00 (reference)
GT	94 (31.8%)	57 (35.8%)	1.22 (0.81–1.84)	0.92 (0.55–1.54)
TT	11 (3.7%)	7 (4.4%)	1.28 (0.48–3.41)	1.79 (0.58–5.51)
GT + TT	105 (35.5%)	64 (40.3%)	1.23 (0.82–1.82)	1.00 (0.61–1.63)
rs4759314				
AA	256 (86.5%)	126 (79.2%)	1.00 (reference)	1.00 (reference)
AG	35 (11.8%)	32 (20.1%)	**1.86 (1.10–3.14)** ***p* = 0.021**	**1.98 (1.03–3.82)** ***p* = 0.042**
GG	5 (1.7%)	1 (0.6%)	0.41 (0.05–3.52)	1.12 (0.09–13.66)
AG + GG	40 (13.5%)	33 (20.8%)	**1.68 (1.01–2.79)** ***p* = 0.046**	**1.92 (1.01–3.64)** ***p* = 0.047**
rs12427129				
CC	240 (81.1%)	128 (80.5%)	1.00 (reference)	1.00 (reference)
CT	53 (17.9%)	28 (17.6%)	0.99 (0.60–1.64)	1.50 (0.80–2.80)
TT	3 (1%)	3 (1.9%)	1.88 (0.37–9.42)	1.40 (0.19–10.56)
CT+TT	56 (18.9%)	31 (19.5%)	1.04 (0.64–1.69)	1.49 (0.81–2.73)

Bold font indicates statistical significance (*p* < 0.05). The odds ratio (OR) and 95% confidence interval (CI) were estimated by logistic regression models. The adjusted OR (AOR) and 95% confidence interval were estimated by multivariable logistic regression models after controlling for age, gender, and tobacco consumption.

**Table 4:** Distribution frequency of the clinical status and *HOTAIR* rs920778 genotype frequencies in 431 urothelial cell carcinoma patients.

Variable	*HOTAIR* (rs920778)			
	TT (%) (*n* = 217)	TC+CC (%) (*n* = 214)	OR (95% CI)	*p*-Value
Stage				
Non-muscle invasive tumor (pTa–pT1)	113 (52.1%)	122 (57%)	1.00 (reference)	
Muscle invasive tumor (pT2–pT4)	104 (47.9%)	92 (43%)	0.82 (0.56–1.20)	0.304
Tumor T status				
Ta–Tcis	50 (23%)	40 (18.7%)	1.00 (reference)	
T1–T4	167 (77%)	174 (81.3%)	1.30 (0.82–.077)	0.267
Lymph node status				
N0	184 (84.8%)	196 (91.6%)	1.00 (reference)	
N1+N2+N3	33 (15.2%)	18 (8.4%)	**0.51 (0.28–0.94)**	**0.031**
Metastasis				
M0	208 (95.9%)	209 (97.7%)	1.00 (reference)	
M1	9 (4.1%)	5 (2.3%)	0.55 (0.18–1.68)	0.295
Histopathologic grading				
Low grade	24 (11.1%)	29 (13.6%)	1.00 (reference)	
High grade	193 (88.9%)	185 (86.4%)	0.79 (0.45–1.41)	0.432

The bold font indicates statistical significance (*p* < 0.05). The odds ratio (OR) and 95% confidence interval (CI) were estimated by logistic regression models.

**Table 5:** Distribution frequency of the clinical status and *HOTAIR* rs12427129 genotype frequencies in 131 urothelial cell carcinoma patients who were smokers.

Variable	*HOTAIR* (rs12427129)			
	CC (%) (*n* = 103)	CT + TT (%), (*n* = 28)	OR (95% CI)	*p*-Value
Stage				
Non-muscle invasive tumor (pTa–pT1)	50 (48.5%)	19 (67.9%)	1.00 (reference)	
Muscle invasive tumor (pT2–pT4)	53 (51.5%)	9 (32.1%)	0.45 (0.19–1.08)	0.074
Tumor T status				
Ta–Tcis	27 (26.2%)	2 (7.1%)	1.00 (reference)	
T1–T4	76 (73.8%)	26 (92.9%)	**4.62 (1.03–20.78)**	**0.046**
Lymph node status				
N0	87 (84.5%)	25 (89.3%)	1.00 (reference)	
N1+N2+N3	16 (15.5%)	3 (10.7%)	0.65 (0.18–2.42)	0.523
Metastasis				
M0	95 (92.2%)	27 (96.4%)	1.00 (reference)	
M1	8 (7.8%)	1 (3.6%)	0.44 (0.05–3.67)	0.448
Histopathologic grading				
Low grade	16 (15.5%)	1 (3.6%)	1.00 (reference)	
High grade	87 (84.5%)	27 (96.4%)	4.97 (0.63–39.19)	0.128

The bold font indicates statistical significance (*p* < 0.05). The odds ratio (OR) and 95% confidence interval (CI) were estimated by logistic regression models.

**Table 6:** Distribution frequency of the clinical status and *HOTAIR* rs12427129 genotype frequencies in 166 UCC patients aged ≤65 years.

Variable	*HOTAIR* (rs12427129)			
	CC (%), (*n* = 128)	CT + TT (%) (*n* = 38)	OR (95% CI)	*p*-Value
Stage				
Non-muscle invasive tumor (pTa–pT1)	67 (52.3%)	21 (55.3%)	1.00 (reference)	
Muscle invasive tumor (pT2–pT4)	61 (47.7%)	17 (44.7%)	0.89 (0.43–1.84)	0.752
Tumor T status				
Ta–Tcis	30 (23.4%)	3 (7.9%)	1.00 (reference)	
T1–T4	98 (76.6%)	35 (92.1%)	**3.57 (1.03–12.44)**	**0.046**
Lymph node status				
N0	106 (82.8%)	33 (86.8%)	1.00 (reference)	
N1 + N2 + N3	22 (17.2%)	5 (13.2%)	0.73 (0.26–2.08)	0.556
Metastasis				
M0	122 (95.3%)	37 (97.4%)	1.00 (reference)	
M1	6 (4.7%)	1 (2.6%)	0.55 (0.06–4.71)	0.585
Histopathologic grading				
Low grade	20 (15.6%)	3 (7.9%)	1.00 (reference)	
High grade	108 (84.4%)	35 (92.1%)	2.16 (0.61–7.71)	0.235

The bold font indicates statistical significance (*p* < 0.05). The odds ratio (OR) and 95% confidence interval (CI) were estimated by logistic regression models.

## Appendix

**Table A1:** Genotype distributions of *HOTAIR* gene polymorphisms among 838 male subjects.

Variable	Controls (*n* = 566), *n* (%)	Patients (*n* = 272), *n* (%)	OR (95% CI)	AOR (95% CI)
**rs920778**				
TT	292 (51.6%)	138 (50.7%)	1.00 (reference)	1.00 (reference)
TC	240 (42.4%)	115 (42.3%)	1.01 (0.75–1.37)	0.95 (0.68–1.32)
CC	34 (6%)	19 (7%)	1.18 (0.65–2.15)	1.41 (0.73–2.73)
TC + CC	274 (48.4%)	134 (49.3%)	1.03 (0.77–1.38)	1.00 (0.73–1.37)
**rs1899663**				
GG	359 (63.4%)	170 (62.5%)	1.00 (reference)	1.00 (reference)
GT	191 (33.7%)	91 (33.5%)	1.01 (0.74–1.37)	0.94 (0.67–1.32)
TT	16 (2.8%)	11 (4%)	1.45 (0.66–3.20)	1.98 (0.81–4.84)
GT + TT	207 (36.6%)	102 (37.5%)	1.04 (0.77–1.40)	1.01 (0.72–1.40)
**rs4759314**				
AA	471 (83.2%)	237 (87.1%)	1.00 (reference)	1.00 (reference)
AG	93 (16.4%)	35 (12.9%)	0.75 (0.49–1.14)	0.79 (0.50–1.24)
GG	2 (0.4%)	0 (0%)	-	-
AG + GG	95 (16.8%)	35 (12.9%)	0.73 (0.48–1.11)	0.77 (0.49–1.22)
**rs12427129**				
CC	451 (79.7%)	222 (81.6%)	1.00 (reference)	1.00 (reference)
CT	114 (20.1%)	48 (17.6%)	0.86 (0.59–1.24)	0.88 (0.58–1.32)
TT	1 (0.2%)	2 (0.7%)	4.06 (0.37–45.05)	6.94 (0.28–169.70)
CT+TT	115 (20.3%)	50 (18.4%)	0.88 (0.61–1.28)	0.91 (0.61–1.36)

**Table A2:** Distribution frequency of the clinical status and *HOTAIR* rs1899663, rs4759314 and rs12427129 genotype frequencies in 431 urothelial cell carcinoma patients.

**Variable**	***HOTAIR* (rs1899663)**			
	**GG (%) (*n* = 265)**	**GT+TT (%) (*n* = 166)**	**OR (95% CI)**	***p*-Value**
Stage				
Non-muscle invasive tumor	141 (53.2%)	94 (56.6%)	1.00 (reference)	
Muscle invasive tumor	124 (46.8%)	72 (43.4%)	0.87 (0.59–1.29)	0.488
Tumor T status				
Ta–Tcis	62 (23.4%)	28 (16.9%)	1.00 (reference)	
T1–T4	203 (76.6%)	138 (83.1%)	1.51 (0.92–2.47)	0.106
Lymph node status				
N0	230 (86.8%)	150 (90.4%)	1.00 (reference)	
N1+N2+N3	35 (13.2%)	16 (9.6%)	0.70 (0.37–1.31)	0.266
Metastasis				
M0	255 (96.2%)	162 (97.6%)	1.00 (reference)	
M1	10 (3.8%)	4 (2.4%)	0.63 (0.19–2.04)	0.441
Histopathologic grading				
Low grade	32 (12.1%)	21 (12.7%)	1.00 (reference)	
High grade	233 (87.9%)	145 (87.3%)	0.95 (0.53–1.71)	0.860
**Variable**	***HOTAIR* (rs4759314)**			
	**AA (%) (*n*=363)**	**AG+GG (%) (*n*=68)**	**OR (95% CI)**	***p*-Value**
Stage				
Non-muscle invasive tumor	199 (54.8%)	36 (52.9%)	1.00 (reference)	
Muscle invasive tumor	164 (45.2%)	32 (47.1%)	1.08 (0.64–1.81)	0.775
Tumor T status				
Ta–Tcis	75 (20.7%)	15 (22.1%)	1.00 (reference)	
T1–T4	288 (79.3%)	53 (77.9%)	0.92 (0.49–1.72)	0.795
Lymph node status				
N0	317 (87.3%)	63 (92.6%)	1.00 (reference)	
N1+N2+N3	46 (12.7%)	5 (7.4%)	0.55 (0.21–1.43)	0.219
Metastasis				
M0	351 (96.7%)	66 (97.1%)	1.00 (reference)	
M1	12 (3.3%)	2 (2.9%)	0.89 (0.19–4.05)	0.876
Histopathologic grading				
Low grade	44 (12.1%)	9 (13.2%)	1.00 (reference)	
High grade	319 (87.9%)	59 (86.8%)	0.9 (0.42–1.95)	0.797
**Variable**	***HOTAIR* (rs12427129)**			
	**CC (%) (*n* = 350)**	**CT+TT (%) (*n* = 81)**	**OR (95% CI)**	***p*-Value**
Stage				
Non-muscle invasive tumor	185 (52.9%)	50 (61.7%)	1.00 (reference)	
Muscle invasive tumor	165 (47.1%)	31 (38.3%)	0.70 (0.42–1.14)	0.150
Tumor T status				
Ta–Tcis	77 (22%)	13 (16%)	1.00 (reference)	
T1–T4	273 (78%)	68 (84%)	1.48 (0.77–2.81)	0.237
Lymph node status				
N0	307 (87.7%)	73 (90.1%)	1.00 (reference)	
N1+N2+N3	43 (12.3%)	8 (9.9%)	0.78 (0.35–1.74)	0.546
Metastasis				
M0	339 (96.9%)	78 (96.3%)	1.00 (reference)	
M1	11 (3.1%)	3 (3.7%)	1.19 (0.32–4.35)	0.798
Histopathologic grading				
Low grade	46 (13.1%)	7 (8.6%)	1.00 (reference)	
High grade	304 (86.9%)	74 (91.4%)	1.6 (0.69–3.69)	0.270

The bold font indicates statistical significance (*p* < 0.05). The odds ratio (OR) and 95% confidence interval (CI) were estimated by logistic regression models.

**Table A3:** Distribution frequency of the clinical status and for *HOTAIR* SNPs frequencies in UCC patients with stratified by age and smoking status.

Variable	Smoker	Non-Smokers	Aged ≤65 Years	Aged >65 Years
	***HOTAIR* (rs920778)**			
Stage	*p* = 0.525 ^a^	*p* = 0.424	*p* = 0.869	*p* = 0.238
Tumor T status	*p* = 0.100 ^b^	*p* = 0.840	*p* = 0.202	*p* = 0.677
Lymph node status	*p* = 0.126 ^c^	*p* = 0.128	*p* = 0.073	*p* = 0.210
Metastasis	*p* = 0.127 ^d^	*p* = 0.665	*p* = 0.275	*p* = 0.710
Histopathologic grading	*p* = 0.263 ^e^	*p* = 0.086	*p* = 0.871	*p* = 0.239
	***HOTAIR* (rs1899663)**			
Stage	*p* = 0.178	*p* = 0.944	*p* = 0.847	*p* = 0.468
Tumor T status	*p* = 0.118	*p* = 0.380	*p* = 0.162	*p* = 0.334
Lymph node status	*p* = 0.317	*p* = 0.544	*p* = 0.589	*p* = 0.310
Metastasis	*p* = 0.134	*p* = 0.354	*p* = 0.604	*p* = 0.574
Histopathologic grading	*p* = 0.236	*p* = 0.303	*p* = 0.736	*p* = 0.595
	***HOTAIR* (rs4759314)**			
Stage	*p* = 0.819	*p* = 0.812	*p* = 0.161	*p* = 0.472
Tumor T status	*p* = 0.769	*p* = 0.872	*p* = 0.599	*p* = 0.479
Lymph node status	*p* = 0.608	*p* = 0.098	*p* = 0.969	*p* = 0.127
Metastasis	*p* = 0.353	*p* = 0.999	*p* = 0.953	*p* = 0.888
Histopathologic grading	*p* = 0.467	*p* = 0.910	*p* = 0.366	*p* = 0.266
	***HOTAIR* (rs12427129)**			
Stage	*p* = 0.074	*p* = 0.611	*p* = 0.752	*p* = 0.087
Tumor T status	***p* = 0.046**	*p* = 0.933	***p* = 0.046**	*p* = 0.761
Lymph node status	*p* = 0.523	*p* = 0.749	*p* = 0.556	*p* = 0.605
Metastasis	*p* = 0.448	*p* = 0.210	*p* = 0.585	*p* = 0.380
Histopathologic grading	*p* = 0.128	*p* = 0.867	*p* = 0.235	*p* = 0.649

The bold font indicates statistical significance (*p* < 0.05). ^a^: The *p*-value was estimated the difference between *HOTAIR* SNPs (homozygous wild-type (WT) alleles and those who had at least one polymorphic allele) and the different stage status (Non-muscle invasive tumor and muscle invasive tumor). ^b^: The *p*-value was estimated the difference between *HOTAIR* SNPs (homozygous wild-type (WT) alleles and those who had at least one polymorphic allele) and the different tumor T status (Ta–Tcis and T1–T4). ^c^: The *p*-value was estimated the difference between *HOTAIR* SNPs (homozygous wild-type (WT) alleles and those who had at least one polymorphic allele) and the different lymph node status (N0 and N1+N2+N3). ^d^: The *p*-value was estimated the difference between *HOTAIR* SNPs (homozygous wild-type (WT) alleles and those who had at least one polymorphic allele) and the different metastasis status (M0 and M1). ^e^: The *p*-value was estimated the difference between *HOTAIR* SNPs (homozygous wild-type (WT) alleles and those who had at least one polymorphic allele) and the different hstopathologic grading status (low grade and high grade).
